# Development of a recombinase-aided amplification method combined with lateral flow dipstick assay to detect *Porcine circovirus* type 2

**DOI:** 10.14202/vetworld.2023.2313-2320

**Published:** 2023-11-19

**Authors:** Ploypassorn Homklinkaew, Sakuna Phatthanakunanan, Siriluk Jala, Alongkot Boonsoongnern, Preeda Lertwatcharasarakul

**Affiliations:** 1 Animal Health and Biomedical Sciences Study Program, Faculty of Veterinary Medicine, Kasetsart University, Bangkok 10900, Thailand; 2 Kamphaeng Saen Veterinary Diagnostic Center, Faculty of Veterinary Medicine, Kasetsart University, Nakorn Pathom 73140, Thailand; 3 Department of Farm Resources and Production Medicine, Faculty of Veterinary Medicine, Kasetsart University, Nakorn Pathom 73140, Thailand; 4 Department of Pathology, Faculty of Veterinary Medicine, Kasetsart University, Nakorn Pathom 73140, Thailand

**Keywords:** lateral flow dipstick assay, *Porcine circovirus* type 2, recombinase-aided amplification

## Abstract

**Background and Aim:**

Porcine circovirus type 2 (PCV2) is a pathogenic virus that suppresses the immune system of pigs, impacting their health and causing economic losses. Rapid diagnostic tools for early detection of PCV2 are critical to disease prevention and control. Several molecular techniques have been established for detecting PCV2 but costly equipment and time-consuming methods are unsuitable for field inspection. In this study, we developed a recombinase-aided amplification combined with lateral flow dipstick (RAA-LFD) assay to compare with polymerase chain reaction (PCR) and quantitative PCR (qPCR) in detecting PCV2 in suspected field samples.

**Materials and Methods:**

To amplify RAA products, 15 primer pairs were designed from the conserved region of the open reading frame (ORF) 1 gene based on multiple alignments of eight PCV2 genotypes. The most efficient primer pair and conditions for the RAA-LFD assay were tested and selected. Limit of detection, repeatability, and reproducibility were determined using the constructed plasmid. DNA was extracted from positive samples for specificity testing as well as from 100 field samples to compare the detection of the RAA-LFD assay with PCR and qPCR.

**Results:**

The F1/R1 primer pair was chosen and labeled with fluorescein isothiocyanate at the 5’ end of the forward primer and with biotin at the 5’ end of the reverse primer. The limit of detection of the RAA-LFD assay was 10 copies/μL at 38°C for 30 min. The RAA-LFD assay was repeatable and reproducible, with no cross-reaction with PCV3, *Actinobacillus pleuropneumoniae*, Porcine epidemic diarrhea virus, Classical swine fever virus, Porcine reproductive and respiratory syndrome virus - North America strain (PRRSV-US) and Porcine reproductive and respiratory syndrome virus - European strain (PRRSV-EU). Based on testing with 100 samples, the developed RAA showed 100% specificity and 90.56% and 85.71% sensitivity when compared to PCR and qPCR, respectively Cohen’s kappa coefficients showed a good agreement with the established techniques.

**Conclusion:**

The RAA-LFD assay targeting the ORF1 gene was highly sensitive, specific, quick, and simple to perform in the field.

## Introduction

Porcine circovirus (PCV) belongs to the genus *Circovirus*, family *Circoviridae*. Recently, PCVs have been classified into four species, including PCV1, PCV2, PCV3, and PCV4 [1–3]. They are all non-enveloped, circular single-stranded DNA. Among them, PCV2 endangers pig health and causes economic losses worldwide in the pig industry [1], which is the primary causative agent of immunosuppressive PCV disease (PCVD) or PCV-associated disease (PCVAD) [4]. Porcine circovirus disease can be categorized by clinical manifestation, including PCV2-associated systemic disease or post-weaning multisystemic wasting syndrome [5]; PCV2-associated reproductive disease (PCV2-RD) [6]; and porcine dermatitis and nephropathy syndrome [7] and subclinical infections. The genome size of PCV2 is approximately 1.7 kb with two main open reading frames (ORFs) [1]. Open reading frame (ORF)1 or *rep* gene encodes the replicase (Rep and Rep’) that are conserved in different genotypes, while ORF2, known as *cap* gene, encodes the capsid protein which is the major structural protein of the virus involved in host immune response and exhibits the greatest sequence variation between various genotypes [8]. In 2018, Franzo and Segalés [9] analyzed the sequences of PCV2 using a phylogeny-grounded genotype and classified the virus into eight genotypes (PCV2a-2h). Genetic diversity studies of PCV2 between 2009 and 2015 showed that the genotypes circulating in Thailand were PCV2a, PCV2b, and PCV2d, with a genotype shift from PCV2b to PCV2d recorded in 2013–2014 similar to happenings worldwide [10]. Interestingly, after 2018, most of the virus genotypes in Thailand were PCV2d, as reported in the Kamphaeng Saen Veterinary Diagnostic Center (KVDC) 2018–2022 annual reports (data not shown). Several molecular detection methods of the virus have been established to ameliorate the economic impact on pig health from PCV2 infection. Polymerase chain reaction (PCR) and quantitative PCR (qPCR) have been widely used in the laboratory for PCV2 detection due to their rapid, highly sensitive, and specific characterization [11–14]. However, these techniques require a thermocycler for amplification, which is costly and inconvenient for field inspection. Isothermal amplification is a technique in which target genes can be amplified at a constant temperature. One well-known technique, loop-mediated isothermal amplification, has also been developed for PCV2 detection without costly tools [15, 16]. Nevertheless, the complexity of primer design and the need for more than 1 h for nucleic acid amplification are still a barrier to field studies. Recombinase-aided amplification (RAA) is an isothermal amplification that uses three major enzymes as recombinase (obtained from bacteria or fungi), single-strand binding protein (SSB), and DNA polymerase [17]. The reaction starts when recombinase forms a complex with a primer called nucleoprotein filament complex and recognizes the complementary base in the DNA template. The double-stranded DNA (dsDNA) template is hybridized with the help of SSB, and a new DNA strand is polymerized by DNA polymerase. This process is repeated continuously to achieve efficient nucleic acid amplification. The reaction can be performed under isothermal condition (37–42°C) within 30 min [18]. This method is rapid, highly sensitive, and specific and easy to perform with no need for costly tools. Several molecular detection methods based on RAA have been developed for both human and animal pathogens such as *Salmonella* spp. [18], hepatitis B virus [19], severe acute respiratory syndrome coronavirus 2 [20], avian infectious bronchitis virus [21], and African swine fever virus [22]. Porcine circovirus type 2 detection methods have been established, with false-negative results in some cases, due to genomic variations among different genotypes [23–25]. Therefore, this study was aimed to develop a test that is applicable to various PCV2 genotypes.

Early diagnosis for the effective prevention and control strategy in pig farms requires developing a simple, rapid, highly sensitive, specific, and visible to the naked eye detection method for PCV2. Combining RAA with the lateral flow dipstick assay allows rapid visual observation without the need for expensive equipment.

In this study, we developed a recombinase-aided amplification combined with lateral flow dipstick (RAA-LFD) assay for PCV2 detection as a simple tool for diagnosing any genotype.

## Materials and Methods

### Ethical approval

This study was approved by the Institutional Laboratory Animal Care and Use Committee of Kasetsart University of Thailand (Permit No. ACKU65-VET-070, 20 September 2022).

### Study period and location

The study was conducted from September 2021 to December 2022 at the KVDC, Faculty of Veterinary Medicine, Kasetsart University, Nakhon Pathom 73140, Thailand.

### Clinical samples

One hundred clinical sera or organs suspected of PCV2 infection were submitted for diagnosis at the KVDC for PCR detection. The DNA/RNA-positive samples of PRRSV-US, PRRSV-EU, CSFV, PEDV, APP, and PCV3 preserved by KVDC were used for specificity testing.

### DNA extraction

Total DNA was extracted from the samples by Indimag48 using IndiMag Pathogen kit (Indical Bioscience, Germany) according to the manufacturer’s instructions, eluted with nuclease-free water in a volume of 40 μL and stored at −20°C until the next step.

### Design of the primers for RAA

Multiple alignments of ORF1 gene sequences from eight genotypes of PCV2 (GenBank: HM038034, MW262923, AF408635, HM038025, HM038022, HM038021, MW262924, AY484407, EU148505, EU148503, KJ094599, KY940534, KY940533, MT188581, KT870146, MG739620, KT369067, MF278779, HQ202972, MG940984, and EU302139) were performed using Bioedit v7. 2 (https://bioedit.software.informer.com/7.2/). The primers were designed based on conserved regions according to the requirements of RAA amplification. The OligoEvaluator™ program (http://www.oligoevaluator.com/LoginServlet) was used to evaluate the chances of dimer and secondary structure. The newly designed primers were listed and located (Table-1).

**Table-1:** Oligonucleotide sequences of the primers used in this study.

Primers	Sequences (5’–3’)	Positions
F1	AACGTTTGTCAGAAATTTCCGCGGGCTGGCTG	423–454
F2	ACTTTTGAAAGTGAGCGGGAAAATGCAGAAGCG	456–488
F3	GGCTGAACTTTTGAAAGTGAGCGGGAAAAT	450–479
F4	GCAGCACCCTGTAACGTTTGTCAGAAATTT	411–440
F5	GTCTACTGCTGTGAGTACCTTGTTGGAGAGCG	357–388
R1	TTCCAGTATGTGGTTTCCGGGTCTGCAAAATTAGC	553–587
R2	TAACAACCACTTCTTCACCATGGTAACCATCCC	608–640
R3	TTCTTCACCATGGTAACCATCCCACCACTT	601–630

*Nucleotide positions refer to the complete genome of the sequence accession number HM038034

### Construction of the recombinant plasmid

The ORF1 gene was amplified by PCR using the newly designed primers. The amplicons were ligated with pLUG-Prime^®^ TA-Cloning vector (Thermo Fisher Scientific, Lithuania) and transformed into JM109 competent *Escherichia coli*.

The ORF1-PCV2-pLUG plasmid was extracted from 37°C overnight well-grown *E. coli* in LB broth with 100 μg/mL ampicillin using Favoprep™ plasmid extraction minikit (Favogen Biotech, Taiwan) following the manufacturer’s instructions. A NanoDrop™ 1000 Spectrophotometer (Thermo Fisher Scientific) was used to measure the concentration of the extracted plasmid and calculate copies using the following equation.

Copy number per unit (copies/μL) = (concentration of DNA (ng) × 6.022 × 10^23^)/(entire length of template × 10^9^ × 650) (dsDNA copy number calculator (uri.edu).

### Polymerase chain reaction

A 10-μL PCR master mix contained 5 μL of Thermo Scientific™ DreamTaq green PCR master mix (2×) (Thermo Fisher Scientific), 0.1 μL of each 10 μM forward and reverse primer, 1 μL of DNA template, and 3.8 μL of distilled water. Porcine circovirus type 2 specific forward primer: PCV2PCR-F (5′-CACGGATATTGTAGTCCTGGT-3′) and reverse primer: PCV2PCR-R (5′-CCGCACCTTCGGATATACTGTC-3′) were described previously [12]. Amplicon product size was 475 bp. The thermal program was: one cycle at 95°C for 3 min; 35 cycles at 95°C for 15 s, 55°C for 30 s, and 72°C for 30 s and one cycle at 72°C for 5 min. Amplicon products were analyzed by 1.5% agarose gel electrophoresis.

### Quantitative polymerase chain reaction

A 10-μL qPCR master mix contained 5 μL of Luna^®^ Universal qPCR master mix (New England Biolabs, USA), 0.25 μL of each 10 μM forward and reverse primer, 0.5 μL of 10 μM probe, 1 μL of DNA template, and 3 μL of distilled water. Porcine circovirus type 2 specific forward primer: PCVqPCR-F (5’-TGGCCCGCAGTATTCTGATT-3), reverse primer: PCV2qPCR-R (5’-CAGCTGGGACAGCAGTTGAG-3’) and probe (6 FAM-CCAGCAATCAGACCCCGTTGGAATG-TAMRA) as described previously were used in this reaction [26]. The thermal program was: One cycle at 95°C for 3 min; 40 cycles at 95°C for 15 s and 55°C for 30 s.

### Optimization of RAA

A 25-μL aliquot of RAA reactions was prepared from RAA nucleic acid amplification kit (Jiangsu Qitian Gene Biotechnology Co., Ltd., China) containing 12.5 μL of Buffer V, 7.9 μL of distilled water, 1.05 μL of 10 μM forward and reverse primer each in a lyophilized master mix, and then 1 μL of DNA template and 1.5 μL of 280 μM magnesium acetate were added. The mixture was incubated at 39°C for 30 min. Then, 25 μL of phenol/chloroform (1:1) was added for post-amplification treatment before centrifugation at 11,300× *g*. The supernatant was analyzed by 2% agarose gel electrophoresis.

The selected primer pair was used to optimize the temperature of the RAA assay, incubated at various temperatures (37–42°C) for 30 min. After the amplification, 25 μL of phenol/chloroform (1:1) was added for post-amplification treatment before centrifugation at 11,300× *g*. The supernatant was analyzed by 2% agarose gel electrophoresis.

### Establishment of the RAA-LFD assay

The selected primer pair was labeled with fluorescein isothiocyanate (FITC) at the 5’ end of the forward primer and with biotin at the 5’ end of the reverse primer for lateral flow dipstick assay.

A 25-μL aliquot of RAA reactions was prepared from RAA nucleic acid amplification kit (Jiangsu Qitian Gene Biotechnology Co., Ltd.) containing 12.5 μL of Buffer V, 7.9 μL of distilled water, 1.05 μL of 5 μM forward and reverse primer each in a lyophilized master mix, and then 1 μL of DNA template and 1.5 μL of 280 μM magnesium acetate were added. The mixture was incubated at 38°C for 30 min. Then, the amplified diluted products with 1% casein in 1 × phosphate buffered saline with 0.05% Tween 20 at ratio 1:100 (1 μL of RAA amplification products and 99 μL of the buffer) were placed in the dipstick tube and results were observed after 10 min.

The dipstick (Serve Science Co., Ltd., Thailand) was used to coat mouse anti-FITC labeled with gold particles on the conjugated pad, mouse anti-biotin monoclonal antibody on the test line, and goat anti-mouse immunoglobulin G on the control line.

### Optimization of the RAA-LFD assay

The RAA-LFD assay was optimized by testing with various primer concentrations, while the optimal time for the RAA assay was also tested by incubation at 38°C for 10, 20, 30, and 40 min. Both results were read out in 10 min by observing the bands appearing on the dipstick.

### Sensitivity and specificity of the RAA-LFD assay

Ten-fold serial dilutions of the ORF1-PCV2-pLUG plasmid were used as a template for the limit of detection of the RAA and RAA-LFD assays. The specificity of the test was confirmed using DNA/RNA from other viruses, including PRRSV-US, PRRSV-EU, CSFV, PEDV, APP, and PCV3 as templates.

### Reproducibility of the RAA-LFD assay

Some concentrations of the plasmid were used as a template to evaluate the repeatability of the developed assay, including 10^6^ copies/μL (strong positive), 10^3^ copies/μL (moderate positive), and 10 copies/μL (weak positive). The test was repeated 3 times. The ORF1-PCV2-pLUG plasmid was serially diluted to test the RAA-LFD assay’s effectiveness after 4 months of storage at −20°C.

### Agreement of the RAA-LFD assay with PCR and qPCR

One hundred clinical samples were tested and RAA-LFD results were compared with PCR and qPCR for sensitivity, specificity, and kappa coefficient.

## Results

### Primer screening and optimization of the RAA reaction

**Figure-1:**
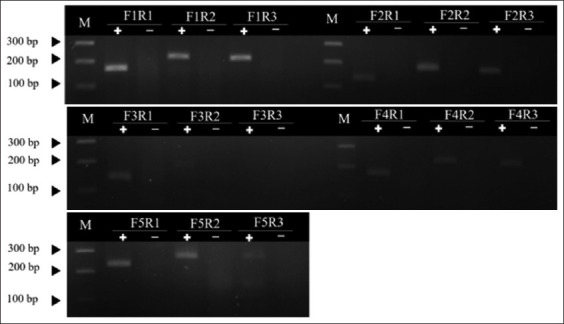
Agarose gel electrophoresis of the recombinase-aided amplification reaction to optimize primer combination. M represents Accuband™ DM2400 (SMOBIO, Taiwan). A plus sign indicates that 10^6^ copies/μL of the plasmid were added. A minus sign indicates no template control.

**Figure-2:**
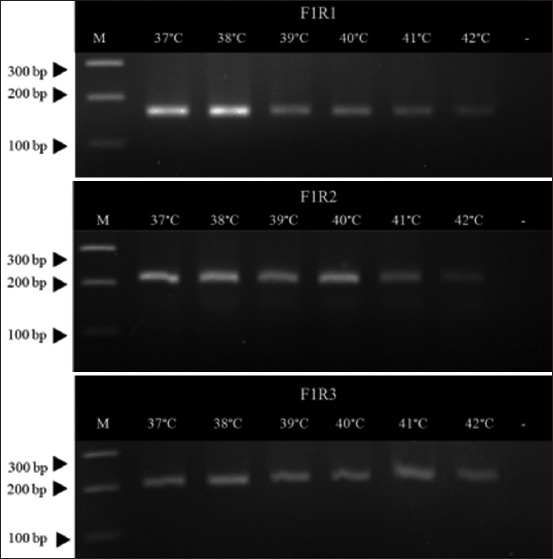
Agarose gel electrophoresis of the recombinase-aided amplification reaction to optimize the temperature for primer pairs F1/R1, F1/R2, and F1/R3. M represents Accuband™ DM2400 (SMOBIO, Taiwan). A minus sign indicates non-template control.

Fifteen pairs of eight newly designed ORF1 gene-specific primers were used for primer screening. The results showed that primer pairs F1/R1, F1/R2, and F1/R3 gave similar strong band intensity. Primer pairs F2/R2, F5/R1, and F5/R2 showed visible bands but not as strong as the former set, while F2/R1, F2/R3, F3/R1, F3/R2, F4/R1, F4/R2, F4/R3, and F5/R3 showed slightly visible bands and F3/R3 showed no band (Figure-1). Based on the similar strong intensity, three primer pairs (F1/R1, F1/R2, and F1/R3) were selected for temperature optimization between 37°C and 42°C according to the manufacturer’s recommendation. The results showed that any temperature could be used for the RAA reaction. However, the DNA bands faded after the temperature was increased. Using F1/R1 primer at 38°C gave the best band result (Figure-2). Therefore, the primer pair F1/R1 was chosen for labeling at the 5’ end of each primer (F1: 5’-FITC-AACGTTTGTCAGAAATTTCCGCGGG CTGGCTG-3’, R1: 5’-Biotin- TTCCAGTATGTGGTTTCCGGGTCTGCAAAATTAGC-3’) and the temperature for the RAA reaction was set at 38°C.

### Optimization of the RAA-LFD assay

**Figure-3:**
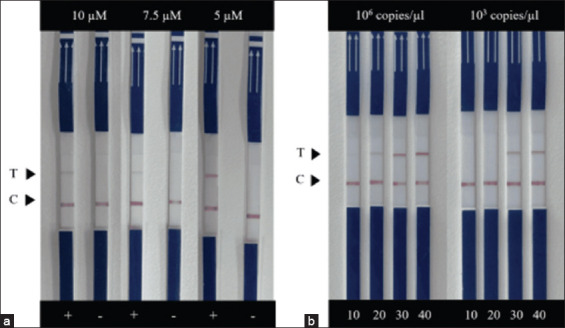
Primer concentration used in the recombinase-aided amplification combined with lateral flow dipstick assay as 10, 7.5, and 5 μM (a). A plus sign indicates that 10^6^ copies/μL of the plasmid were added. A minus sign indicates no template control. Optimizing times for the recombinase-aided amplification reaction with 10^6^ and 10^3^ copies/μL of the plasmid added as a template. (b) The reactions were incubated at 38°C for 10, 20, 30, and 40 min, respectively. C and T represent control and test lines, respectively.

The results showed that the optimal primer concentration used for RAA-LFD was 5 μM for each because that gave the most obvious test line (Figure-3a). Time optimization used 10^6^ copies/μL of the plasmid to represent strong positive and 10^3^ copies/μL for weak positive. The results showed that a weak positive was detected within 30 min of the RAA reaction (Figure-3b).

### Sensitivity and specificity of the RAA-LFD assay

**Figure-4:**
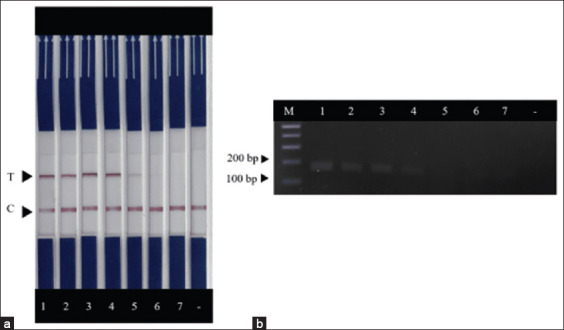
Limit of detection of the recombinase-aided amplification combined with lateral flow dipstick assay. Positive products (164 bp) were detected by lateral flow dipstick (a) and 2% agarose gel electrophoresis (b). Lanes 1–7 represent the plasmid concentrations of 10^6^-1 copies/μL, respectively. A minus sign represents non-template control. C and T represent control and test lines, respectively.

**Figure-5:**
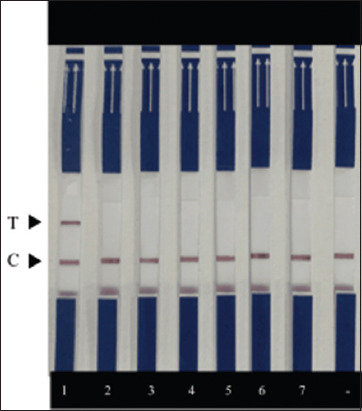
Specificity of the PCV2 recombinase-aided amplification combined with lateral flow dipstick assay. Other viruses symbolized as lanes 1–7 including PCV2, PCV3, APP, PEDV, CSFV, PRRSV-US, and PRRSV-EU, respectively. A minus sign represents non-template control. C and T represent control and test lines, respectively. PCV=*Porcine circovirus*.

The minimum detectable concentration using the RAA assay was 10^3^ copies/μL (Figure-4b). However, when combined with the lateral flow dipstick assay the sensitivity improved to 10 copies/μL (Figure-4a). The specificity of RAA-LFD was tested by investigating the cross-reaction between PCV2 and other viruses including PRRSV-US, PRRSV-EU, CSFV, PEDV, APP, and PCV3. Results showed no cross-reaction between these viruses (Figure-5).

### Repeatability and reproducibility of the RAA-LFD assay

**Figure-6:**
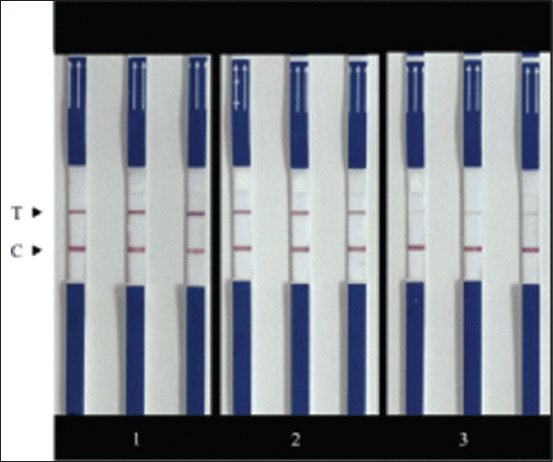
Repeatability of the *Porcine circovirus* type 2 recombinase-aided amplification combined with lateral flow dipstick assay. Lanes 1–3 including strong positive, moderate positive, and weak positive, respectively. C and T represent control and test lines, respectively.

The readability of three repeated RAA-LFD assays was evaluated as strong positive, moderate positive, and weak positive. The repeated strong and moderate positive results were read out at an intensity similar to that of the test line. The intensity of the test line in weak positive repetition showed a slight difference, but all three repeated assays were read as positive (Figure-6).

The reproducibility was tested by repeating the sensitivity assay 4 months after the first trial. The sensitivity of the RAA-LFD assay still showed 10 copies/μL.

### Clinical sensitivity and specificity of the RAA-LFD assay

The performance of PCV2 RAA-LFD was evaluated using 100 clinical samples. When comparing RAA-LFD with conventional PCR and qPCR, results showed that 53 (53%) samples were positive and 47 (47%) samples were negative by conventional PCR, but 56 (56%) samples were positive and 44 (44%) samples were negative by qPCR. However, only 48 (48%) of the samples were detected by RAA-LFD, while 52 (52%) gave a read out as a negative result. Some samples gave results from the three methods that were not agreeable as false-negative and false-positive, as shown in Table-2.

**Table-2:** Comparison of positive and negative results among the PCV2 RAA-LFD, conventional PCR, and qPCR.

RAA-LFD	PCR			qPCR		
	
	Positive	Negative	Total	Positive	Negative	Total
Positive	48	0	48	48	0	48
Negative	5	47	52	8	44	52
Total	53	47	100	56	44	100

PCR=Polymerase chain reaction, qPCR=Quantitative polymerase chain reaction, RAA-LFD=Recombinase-aided amplification combined with lateral flow dipstick

The sensitivity, specificity, and kappa coefficient were 90.56%, 100%, and 0.90, respectively, when comparing the RAA-LFD assay with conventional PCR and 85.71%, 100%, and 0.84 when comparing with qPCR.

## Discussion

This study developed a PCV2 detection kit that was rapid, instrument-free, easy to use, portable, and highly sensitive and specific. The RAA technique with high sensitivity and specificity was chosen [18, 20]. Similar to the recombinase polymerase amplification (RPA) technique, RAA was performed using various techniques such as agarose gel electrophoresis or fluorescence detection [27]. However, when using these two methods, extra equipment that is unsuitable for field inspection is required. The combination of RAA with a dipstick was read out by the naked eye, did not require expensive instruments, and was suitable for field use.

Recombinase-aided amplification combined with a dipstick is recommended for use with a specific probe [21–23] but probe modification is challenging and expensive. Therefore, labeling both primers instead of probe synthesis was chosen as established previously in RPA [28].

Primers specific for the conserved regions of PCV2 ORF1 gene were newly designed. The previous studies by Chen *et al*. [23], Yang *et al*. [24], and Wang *et al*. [25] used the RAA or RPA techniques to detect PCV2 focused on the ORF2 gene. According to genetic diversity, the ORF1 gene is more conserved among PCV2 genotypes than the ORF2 gene. The PCV2 ORF2 encodes the major structural capsid protein which exhibits a higher rate of variation for viral capsid modification in tissue tropism or virus-host interactions [29]. Among 15 pairs of new ORF1 new designed primers, results showed that primer set F1/R1 was the most efficient. The RAA reaction was performed in the range 37–42°C, complying with the manufacturer’s recommendation, using a slightly changed temperature device such as a water bath. The optimal condition was 38°C for 30 min.

The manufacturer recommended using 10 mM of each primer for the RAA-LFD assay but the dilution test line was much clearer at a concentration of 5 μM each. A reduction of primer dimer made the labeled amplicon more easily detected at the test line.

The limit of detection of the RAA-LFD assay was 10 copies/μL which was 10 times higher than previously published results [23, 24]. The newly designed primer pair was more sensitive to the target PCV2 ORF2 gene. No detailed suggestions for primer design have been previously reported so the manufacturer’s recommendations for primer selection are still needed. In this study, no cross-reaction was found with PCV3, APP, PEDV, CSFV, PRRSV-US, and PRRSV-EU, while multiple alignments of the new design primer sequences with eight genotypes of PCV2 showed that they were in the conserved regions (data not shown), supporting that this new PCV2 RAA-LFD kit could be used for any genotype of PCV2.

The developed RAA-LFD was repeatable even with a weak positive sample. The reproducibility test showed that the enzyme could still work for at least 4 months after first use if stored at −20°C. According to the manufacturer, RAA shelf-life was 12 months with evidence that weak positivity could still be detected after 1 year [22].

One hundred clinical samples were tested to compare the RAA-LFD assay with conventional PCR and qPCR and there were still some false-negative results. Most of the false-negative samples had Cq values >33 which could indicate weak positive samples (data not shown). The previous studies [30, 31] also found that the newly developed RAA or RPA had some false-negative values compared to PCR or qPCR, even targeting the same gene, with the newly developed techniques slightly less sensitive than the reference technique. However, the overall agreement was still acceptable [30, 31]. Cohen kappa coefficients of this RAA-LFD with PCR and qPCR were 0.9 and 0.84, respectively, indicating almost perfect agreement. Porcine circovirus type 2 can be found in healthy pigs. The virus can circulate in pig farms without any PCVAD signs and evidence suggested that no healthy pigs had viral loads >10^6^ PCV2 genomes per mL serum [32, 33]. The aim of PCV2 detection in field use is to confirm the existence of the virus. For this purpose, a very highly sensitive test is not as essential as a field practical test.

Taken together, the main advantages of the newly developed RAA-LFD include the ability to detect any PCV2 genotypes, quick detection that saves time, and easy to perform with results visualized by the naked eye using an instrument-free technique that is suitable for field use.

## Conclusion

In this study, we developed a sensitive, specific, and rapid PCV2 detection method using a conserved region of the ORF1 gene. Consequently, the assay was detectable in any PCV2 genotype. The combination of the RAA and lateral flow dipstick assays did not require many instruments, was suitable for field inspection, and was easy to perform.

## Authors’ Contributions

PH and PL: Designed and supervised the study and drafted and revised the manuscript. SP: Preparation of gene constructs and PCR and qPCR of the samples. SJ: Optimized the RAA-LFD assays. AB: Collected samples and supervised the study. All authors have read, reviewed, and approved the final manuscript.
